# Identification of Population Affinity Using Dental Traits: A Narrative Review in Forensic Odontology

**DOI:** 10.1002/hsr2.71514

**Published:** 2025-11-18

**Authors:** Alok Atreya, Ritesh G. Menezes, Srinivasa Rao Bolla, Samarika Dahal

**Affiliations:** ^1^ Department of Forensic Medicine Lumbini Medical College Palpa Nepal; ^2^ Forensic Medicine Division, Department of Pathology, College of Medicine Imam Abdulrahman Bin Faisal University Dammam Saudi Arabia; ^3^ Department of Biomedical Sciences, School of Medicine Nazarbayev University Astana Kazakhstan; ^4^ Department of Oral Pathology and Forensic Dentistry Tribhuvan University Institute of Medicine Maharajgunj Medical Campus Kathmandu Nepal

**Keywords:** ancestry, Carabelli's cusp, forensic odontology, odontometry, population affinity

## Abstract

**Background and Aim:**

Forensic odontology utilizes dental morphology to estimate population affinity in medico‐legal cases. This review examines the role of dental traits in human identification, emphasizing their limitations in conclusively estimating ancestry or ethnicity.

**Methods:**

A comprehensive search was conducted across databases such as PubMed, ScienceDirect, Scopus and Web of Science using key terms ‐ race, ancestry, ethnicity, population affinity and forensic odontology. Literature studying race/ancestry (older studies) and population affinity (newer studies) was reviewed.

**Results:**

All dental features are characterized by a certain degree of variation across human populations. On one hand, tooth characteristics may seem useful for identifying ancestry and ethnicity. Yet, combining dental traits with other evidence may improve ancestry estimation. Training observers with additional sources and practical skills improves accuracy and produces more reliable results. The observational method, while systematic, risks oversimplifying contextual factors. However, in forensic odontology, the broader social context of this approach must be considered, as it evaluates whether race is a biological notion.

**Conclusions:**

Traditional terms like race and ancestry should be replaced by population affinity. Future recommendations include developing improved methods to enhance result accuracy.

## 1 Introduction

Forensic odontology applies dental morphology to help in medico‐legal investigations, such as the estimation of population affinity using dental characteristics. These characteristics are influenced by genetic and environmental factors and vary widely across human populations [1]. Although anthropologists assert that race is a social construct rather than a biological classification [2], many studies continue to use traditional racial classification: Caucasoid, Negroid, and Mongoloid [3, 4].

The labelling of human populations as ‘Caucasoids’ (Whites), ‘Negroids’ (Blacks or African Americans), and ‘Mongoloids’ (split into Asians and Southwest Mongoloids) has been typical of the older historical studies. Scientific advancements meanwhile, have made those terms which originated from early anthropological classifications archaic and socially problematic because they connect to the debunked notions of biological race. Today's anthropologists and geneticists favor the term ‘ancestry’ instead of ‘race’ [2]. Ancestry is complex and imprecise [5]. For human identification purposes, forensic anthropologists support a transition away from classification by race/ancestry, but by ‘population affinity’ instead. The terminologies, ‘population’ refers to groups that share genetic and environmental characteristics; ‘ancestry’ denotes genetic heritage; ‘population affinity’ describes shared biological characteristics independent of race; and ‘race’ is a social construct without biological validity. According to the Scientific Working Group for Forensic Anthropology (SWGANTH), population affinity is what other biological profiles; for example: age and sex; rely on in forensic identification. Yet, factors like evolutionary processes and genetic factors, transatlantic migration/trade of slaves, economic and political migrations, intermarriage, socio‐political influences, and transnationalism, complicate such mappings, rendering it difficult to make neat biological and social distinctions [6]. Therefore, contemporary forensic anthropologists prefer the expression of ‘population affinity’ to the use of terms such as ‘ancestry’ and ‘race’ [6].

Anthropologists repudiate the concept of biological race, postulating that human variation is gradual and clinal, not limited to distinct racial categories [7]. The American Association of Physical Anthropologists (AAPA) asserts that race, a social defined category inseminated by European colonists to justify domination, has no biological significance given that humans share 99.9% of their genome in common, and phenotypic traits, including dental morphology vary gradually across populations [7]. Rather, forensic odontology should employ the ‘population affinity’ to refer to common biological characteristics, recognizing the dynamic interplay of genetic and phenotypic variations involving migration, gene flow, and environmental factors. Yet, racism, through racial ideologies, has real biological impacts, that affect health and well‐being through structural inequities [7]. This review uses historical terms like ‘Mongoloid’, ‘Caucasoid’, and ‘Negroid’ only when referencing older literature, prioritizing population affinity to avoid perpetuating racial biases.

Notwithstanding ongoing disputes over terminology, dental traits remain pivotal in forensic identification. Dental morphology offers clear evidence for estimating population affinity [1]. Nevertheless, the usefulness and validity of these methods need careful assessment in modern forensic contexts that may include mixed populations.

Therefore, this review aimed to explore dental traits unique to populations. It also addresses current knowledge, limitations, and challenges associated with dental trait analysis in forensic odontology.

## 2 Methods

This narrative review addresses the research question: “What is the current knowledge in forensic odontology related to the identification of ancestry and ethnicity using dental traits, including the reliability and limitations of dental analysis, and future research directions?” To address this a comprehensive search was conducted across databases such as PubMed, ScienceDirect, Scopus and Web of Science using key terms ‐ race, ancestry, ethnicity, population affinity and forensic odontology. Citation tracking was done for historical or notable studies. Grey literature such as report and thesis was also included. Literature studying race/ancestry (older studies) and population affinity (newer studies) was reviewed.

## 3 Dental Complexes Associated With Population Affinity

The study of dental morphology for population affinity estimation in forensic odontology has progressed since the 19th century, evolving from early anatomical observations to systematic, population‐based analyses [8]. Early work by Owen (1845) and von Carabelli (1842) identified distinctive dental traits such as Carabelli's cusp, which established the basis for odontology and dental anthropology [8, 9]. Study on shovel‐shaped incisors introduced a quasi‐continuous scale and associated specific traits with populations, notably linking shovel‐shaped incisors to Asian and Native American groups [9]. By the mid‐20th century, dental complexes were categorized to describe population‐specific traits, but these terms are now obsolete because of their racial connotations [10, 11]. Hanihara [10] introduced the Mongoloid, Caucasoid, and Negroid dental complexes; which are now outdated; to describe population affinities on the basis of dental traits. These historical categories, based in colonial racial ideologies, meanwhile ignore the continuous nature of human biological diversity and its disconnection from socially defined racial groups [7]. Modern forensic odontology employs standardized systems, such as the Arizona State University Dental Anthropology System (ASUDAS), to analyze global trait frequencies for population affinity estimation that acknowledges continuous human variation [8, 12].

### 3.1 Mongoloid Dental Complex

Mongoloid dental complex comprises six primary crown morphologies that occur regularly in Mongoloid populations [10, 13]. These include shovel‐shaped upper central (UI1) and lateral incisors (UI2), a deflecting wrinkle in deciduous second molar (dm2), protostylid on lower first molars (LM1), seventh cusp on the lower second molar (LM2), and a metaconule on the upper second molar (UM2) [10, 13]. Hanihara [11] later excluded the seventh cusp due to its low frequency in Malaysians [11, 14]. To contextualize dental variation in Malaysia, reference may be made to Tratman (1950) as cited by Khamis [14] which is an early record of research on dental morphology in the country, where Tamils (Indo‐European) and Chinese/Malays (Mongoloid) were compared [14]. Tratman identified Mongoloid traits such as shoveling, dens evaginatus, double shovel, enamel extension (90% prevalence), taurodontism, and sixth cusp on the lower first molar (LM1), and Indo‐European traits like Carabelli trait [14]. While it is historical in nature given the World War II data loss, Tratman's work is relevant to forensic odontology as it documented population specific traits which can be useful in the identification process in the diverse population of Malaysia [14]. It is not the first dental morphology study in the world, as 19th‐century work predated it, but it is the pioneer for Malaysia, serving as a reference for Khamis' [14] statistical analysis, which shows shoveling to be present among Mongoloids but Carabelli trait to be of mild frequency in Indians. While Khamis' citation illustrates this historical continuum, it challenges Tratman's observation to improve forensic estimate of ethnicity [14].

Tratman's data corroborate Hanihara's [10] description of Mongoloid dental complex, rather than justify it [10, 14, 15].

#### 3.1.1 Sinodonts and Sundadonts

Turner [16] categorized populations that had traditionally been called ‘Mongoloid’ into two dental patterns: Sinodonts (Northern Asian and Native American population) and Sundadonts (Southeast Asian population) [16]. The study identified 28 dental traits in several East Asian populations, including prehistoric and recent samples [16]. Turner used the mean measure of divergence to evaluate the biological distance within East Asian people and presented the resulting matrix in cluster analyses [16]. Furthermore, Turner's study also revealed that the Southeast Asian (Sundadont) cluster could be segregated into two minor clusters [16]. The first minor cluster includes individuals from Nepalese, Filipinos, East Malay Archipelago, lndomalaysians, and Burmese ancestry, while the second includes those of prehistoric Taiwanese, Thai, early Mainland Southeast Asian, early Malay Archipelago, and recent Southeast Asian (Indonesian) ancestry [16]. Turner [16] proposed that Sundadont dental pattern in Southeast Asian populations, including Malays, resulted from admixture with Indian Caucasoid populations or cultural influences from Arab and Indian traders, missionaries, and colonists [16]. A conclusive explanation emerged from comparing an Asiatic Indian sample with Malays and Chinese, confirming distinct dental traits like shoveling in Malays and Carabelli trait in Indians [14]. Furthermore, Turner noted that several dental traits such as UI1 shovelling, UI1 double shovel, LM1 deflecting wrinkle, UM1 enamel extension, UM3 peg/reduced/congenital absence, LM1 three roots, LM2 four cusps, and UP1 one root had significant differences in mean frequency between Sinodonts and Sundadonts [16].

#### 3.1.2 Proto‐Sundadont

Hanihara [13] identified five dental traits distinguishing the three fundamental divisions of the Mongoloid dental complex as follows‐ Sundadonty, Sinodonty, and the dental pattern of Australian Aborigines [13]. The Australian aboriginal dental pattern demonstrates evolutionarily conservative features that conform to a “proto‐Sundadont” pattern, which might be a transitional precursor before Sundadonty and Sinodonty had evolved [13]. Australians and Southeast Asian Negritos, likely the indigenous inhabitants of Southeast Asia, possibly shared a common ancestral origin, with Negritos subsequently developing the more advanced Sundadont dental pattern [13].

#### 3.1.3 Super‐Sinodont

Stojanowski et al. [17] found that Native American dental morphology does not fit either of Turner's [16] Sinodont and Sundadont complexes, indicating a unique pattern [17, 18]. This pattern with high shovelling, extra cusps, and complex occlusal surfaces indicates a rugged tooth appearance. Scott et al. coined the term “super‐Sinodont” to describe this pattern [19]. Although there is variability, significant dental morphological differences are seen between Asian and Native American populations [18]. More so than in Asian Sinodont and Sundadont populations, Native Americans are characterized by enhanced dental trait expressions, resulting in strong forensic distinctions between Asian and New World populations [20]. This super‐Sinodont model, that is most likely the consequence of the prolonged isolation of ancestral Native Americans during Beringian standstill, maintains the Northeast Asian dental relationships but acquires novel traits [20]. Recent findings support the pervasiveness of super‐Sinodonty in ancient and modern Native American populations, highlighting its significance for understanding dental evolution and forensic identification [21].

### 3.2 Caucasoid Dental Complex and Eurodont

Mayhall et al. (1982) studied dental casts of American white people and defined what was historically termed as ‘Caucasoid dental complex’ of the permanent dentition [22]. This complex is characterized by certain traits, such as the absence or only a trace shoveling, no bilateral winging, no premolar occlusal tubercles or odontomes, and Carabelli's trait expressed as a cusp or bulge [22]. The complex also includes rare or absent LM1 protostylid, LM1 cusp 6, and cusp 7 [22].

Later on, the dental features of populations from Europe, the Middle East, North Africa, and India were also studied [22]. The term ‘Caucasoid’ is outdated and has been replaced by ‘Eurodont’ in modern forensic odontology to describe dental variation seen in Western Eurasia [22]. Eurodont dental pattern includes minimal incisor shoveling, double‐shoveling, and winging, and UI2 interruption grooves of moderate frequency. Lower molars (LM) are characterized by the highest frequency of four‐cusped LM1 and LM2 and the lowest frequencies of LM1 cusps 6 and 7. The deflecting wrinkle frequency is unusually high in the living Basque sample [22].

The teeth of the Basque people slightly differ from those of other Europeans, likely due to genetic and historical factors [22]. Most modern European populations today have a genealogy which is majorly based on the European Palaeolithic, with a small influence of Neolithic. The Basques could be an example of a population that has retained some of these ancient traits [22].

### 3.3 Sub‐Saharan Dental Complex and Afridonty

Irish (1993) coined “Sub‐Saharan African Dental Complex” (SSADC) to describe the dental morphology of Sub‐Saharan Africans [23]. Historically, such populations were sometimes labelled as ‘Negroid’ in early anthropological literature.

The SSADC, also referred to as ‘Afridonty’ pools diverse samples of sub‐Saharan populations from the nineteenth to early twentieth centuries [23]. The SSADC includes a suite of 11 traits that differentiate sub‐Saharan people from other samples worldwide [23]. The high‐ and low‐frequency traits that best characterize the greater sub‐Saharan African population pattern are UC Bushman canine, two‐rooted UP1, UM1 Carabelli's trait, three‐rooted UM2, LM2 Y‐groove pattern, LP1 Tome's root, two‐rooted LM2, and UM3 presence [23]. In addition, they share among the lowest frequencies of UI1 double shoveling and UM1 enamel extension. While individual traits are not uniquely important, the appropriate combination of all 11 traits clearly identifies the sub‐Saharan African pattern.

### 3.4 Australoid Dental Complex

Studies of Aboriginal Australian populations have identified dental traits historically termed ‘Australoid dental complex’, including a high frequency of the entoconulid trait, most common on third molars [24]. Its degree of expression is 80% for the deciduous second molars (dm2) with small cusps, 50% on permanent second molars (M2), 70% for the permanent first molars (M1), and 80% on permanent third molars (M3) [24]. Other common dental traits are the expression of metaconules and Carabelli trait on maxillary molars (UM), and the sixth cusps and protostylids on mandibular molars (LM) [24]. It seems that these dental traits depend on both genetic and environmental factors [24].

## 4 Dental Morphology

Instead of categorizing the human population into outdated racial groups, dental anthropologists have subdivided humankind into four regions: Western Eurasia, Sub‐Saharan Africa, Sino‐Americas, and Sunda‐Pacific [23, 25]. Western Eurasia covers all of Europe and parts of Africa and Asia. Populations in this subdivision inhabit regions such as peninsular Europe, the British Isles, North Africa, and much of the Indian subcontinent. The major language families represented in this subdivision are Indo‐European, Afro‐Asiatic, Caucasian, and Uralic. Sub‐Saharan Africa includes populations referred to as Negroid or Ethiopian. The most widely distributed populations in Africa fall within the Niger‐Kordofanian phylum. Sino‐Americas include populations commonly known as Mongoloids or proto‐Mongoloids. They share many biological and linguistic features, and Joseph Greenberg reduced New World languages to three primary families: Amerind, Na‐Dene, and Eskimo‐Aleut [26]. Sunda‐Pacific refers to Mainland Southeast Asia, islands of the East Indian Archipelago, and populations of Polynesia within the Austronesian language family. Sahul‐Pacific includes Australia, New Guinea, and Tasmania, and the people of Australia show no linguistic ties to any population outside the continent. Remains found in different regions of the world may use different reference populations to estimate population affinities, designed for specific geographic and demographic context.

Based upon the morphological dental traits highlighted in the studies from Scott & Turner (1997) [20], Irish (1993), and Bailey [27] the following morphological traits of the teeth are identified to distinguish between populations of various regions (Table 1) [25, 28, 29].

**Table 1:** Morphological traits of the teeth to distinguish between the populations from various regions.

	Worldwide	High frequency	Intermediate frequency	Low frequency
Labial convexity	0% to 41.7%	South Asia (India), sub‐Saharan Africa		North Asia, Sahul‐Pacific
Shoveling UI1	3% to 85.8%	North Asia, Americas		Western Eurasia, Sahul‐Pacific
Double shoveling UI1	0% to 70%	Native Americans, North Asia	East Asia, American Arctic	Western Eurasian, sub‐Saharan African, Sunda‐Pacific, Sahul‐Pacific
Tuberculum dentale UI2	0% to 50%	South Asia (India)	Sub‐Saharan Africa	North Asia
Canine mesial ridge (Bushman canine)	0% in non‐African sample; 0% to 40.6% in sub‐Saharan African sample	Sub‐Saharan Africa		
Canine distal accessory ridge	20% to 75%	Shaul‐Pacific		South Asia
Carabelli′s trait UM1	1.9% to 30.1%	Western Eurasia		Sino‐America
Cusp 5 UM1	20.6% to 88.2%	Sahul‐Pacific, Austral Asia,	Sunda‐Pacific, Sub‐Saharan Africa	Western Eurasia, Sino‐America
Three‐cusped upper molars UM2	0% to 33%	Western Eurasia		Sub‐Saharan Africa
Lingual cusp number LP2	37.5% to 87.5%	Shaul‐Pacific		Western Europe, South Asia
U‐shaped fissure LP2	23.3% to 75%	Sahul‐Pacific		Sub‐Saharan Africa
Middle trigonoid crest LM1	0% to 5%	South Africa, Southern Africa		
Cusp 6 LM1	1.9% to 52.3%	Sunda‐ and Sahul Pacific	Sub‐Saharan Africa	Western Eurasia
Cusp 7 LM1	1.6% to 42.9%	Sub‐Saharan Africa		Rest of the world
Four‐cusped LM1	0.0% to 19.1%	Western Eurasia, Sahul Pacific		Africa, Sino‐America
Four‐cusped LM2	~100%	Western Eurasia		Sub‐Saharan Africa
Deflecting wrinkle LM1	5.6% to 55.5%	Sino‐America, Southern Africa		Western Eurasia
Y‐pattern LM2	1.5% to 68.7%	Sub‐Saharan Africa		Sino‐America
Premolar shape LP2	Rare			
Transverse crest LP2	Rare (single case reported for each North Asia, South Asia, and Sahul Pacific region)			

### 4.1 Shovel Shaped Incisors

Shovel‐shaped incisors have mesial and distal marginal ridges on the lingual surface of the upper and lower anterior teeth [28], and probably have been scrutinized more than any other dental trait in ancestry determination. These ridges are more frequent among Asian and Native American populations and infrequent or absent among African and European populations [28]. The development of various scales for grading incisor shoveling is discussed, and particular attention is given to the scale defined by Hrdlicka, which now serves as the standard nomenclature [28, 30]. Shoveling is most frequently expressed on the upper incisors, however, it can also be observed on the lower incisors and canines [30]. The prominence of the essential lingual lobe on the upper canines prevents the formation of a fossa, which is the hallmark of shoveling [30]. Scott and Turner (1997) noted that populations with frequent shoveling also tend to have high rates of double‐shoveling [30]. Shovel‐shaped incisors are critical for estimating ancestry. A significant correlation is observed between total trait frequency and degree of expression, with Native Americans manifesting the most pronounced expressions of the trait [30].

### 4.2 Carabelli's Trait

Carabelli's trait, also known as Carabelli's cusp or tubercle of Carabelli, appears on the mesiolingual or lingual aspect of upper molars, particularly UM1 [30]. This trait shows a wide variation, from small ridges and grooves to large tubercles with free apexes [30]. In 1956, Dahlberg introduced a rating scale for the trait, which includes both its absence and seven grades that indicate the size and number of features [30]. These ranged from minor grooves and ridges to large free‐standing cusps [30]. Although some researchers warn against a heavy reliance on Carabelli's trait for ancestry estimation, the consideration of this dental feature in combination with other traits still allows us to gain an understanding of general population patterns [28].

While Carabelli's trait was once thought to be unique to Europeans, it has been found in other populations as well [30]. Different populations exhibit varying frequencies and expression of this trait, making it unsuitable for ancestry estimation on its own. However, Carabelli's trait correlates with other dental traits such as protostylid, hypocone, and tooth size, which can aid in ancestry estimation when considered together.

## 5 Odontometrics

Tooth morphometry has been used to identify population affinity. For example, Henry Fowler created a dental index to divide human population affinity into micro‐, meso‐, and megadonts [31]. Microdontic, mesodontic, and megadontic refer to tooth size categories based on tooth crown measurements. Microdontic teeth are smaller than average, mesodontic teeth are of average size, and megadontic teeth are larger than average. Maximillian de Terra suggested that tooth size was in proportion to body size and could not be used to distinguish between population affinities [31]. Miyabara was able to distinguish Japanese teeth from European teeth by their crown sizes [31]. Later studies using statistical techniques found ethnic differences in tooth size, with some populations having larger or smaller teeth than others [31].

A study was conducted to compare the size of teeth between four different groups: Southern Chinese, North American of European ancestry, Modern British of European ancestry, and Romano‐British; using mesio‐distal crown dimensions as the primary variable [32]. The study found that the size of teeth measured using hand‐held digital calipers on dental cast varied between the different groups, with the Southern Chinese having the largest teeth and the Romano‐British having the smallest [32]. However, tooth size varied among groups for each tooth type [32]. Hand‐held digital calipers when used to obtain measurements from the cast, may not provide the most accurate measurements compared to modern techniques such as 2D and 3D imaging [33]. Additionally, the study only included specific populations, and the results may not be generalizable to other populations or ethnic groups [32].

A study conducted in India upon tooth crown metric dental traits found that among four ethnic groups Hindus, Muslims, Christians and Iranians, Christian sample was found to have the largest teeth overall, whereas the Iranian sample generally displayed the smallest mesiodistal and buccolingual crown dimensions [34].

A study of Caucasoid, Australoid and Mongoloid populations found that males exhibited a large crown size than females [35]. Furthermore, Australian Aboriginals were megadontic who had the largest dental crown size in both mesiodistal and buccolingual dimensions [35].

However, there are limitations to utilizing tooth morphometry for estimation of population affinity. Early studies often employed univariate statistics such as Student's *t*‐tests, which restrict comprehensive analysis of intergroup differences [31]. While multivariate statistics enable more robust assessments, they also risk misconceptions and overdependence on complex statistical methods [31].

Moreover, we cannot accurately establish the human population group membership by using only dental metrics because the tooth size and form vary within ethnic groups due to genetic and environmental factors. Therefore, the tooth size for different groups still largely overlaps and thus cannot be confidently used. Instead, a combination of diverse physical attributes and genetic markers should inform population affinity estimates.

Burris and Harris [36, 37] conducted studies to differentiate American Blacks and Whites using palate dimension and maxillary arch size [36, 37]. They reported that racial classification was 83% accurate using palate dimension [36]. American Blacks exhibit 10% wider and 12% deeper maxillary arches than Whites, with a squarer form (palatal index: 72.6 vs. 69.3, *p* = 0.0002 [37]. Likewise, palatal dimension was utilized to differentiate Euro‐Americans, Afro‐Americans, and Amerindians [38]. Maxillary arch metrics and palatal dimension have a potential of being forensic marker for population affinity estimates which should further be explored.

## 6 Cultural Practices Affecting Teeth

### 6.1 Dental Modification

Intentional dental modifications, practiced globally, reflect cultural, aesthetic, and social values of the community [39]. In the Caribbean islands, such practices emerged during colonial era, often linked to African traditions and subsequently adopted by the African people and their descendants throughout the world [40]. Roksandic et al., report that remains of the pre‐European individual E‐105 from Cuba, showing deliberate crown reduction, could be indicative of the oldest known case of dental modification in the Antilles, predating African influence [40]. Similar patterns in six other individuals match post‐contact African and African‐descendant Caribbean practices [40]. In pre‐Columbian Costa Rica (1000–1300 CE), filing and inlay preparations (e.g., incisal notching, angle modifications) in the Gran Nicoya region suggest elite status and cultural identity [41]. Globally, modifications like avulsions (e.g., Dinka tribe, Sudan) and inlays (e.g., ancient Mexicans) signify rites of passage or status [39]. These practices aid forensic identification of ethnic origins [39, 41].

### 6.2 Dental Chipping

Dental chipping, or microfractures of the tooth crown reveal pattern of microtrauma in European and Arctic population that provide insights into dietary and tooth‐tool use behavior [42]. Three samples from St. Lawrence Island Inuit, medieval Norwegians, and medieval and postmedieval Spanish were examined [42]. The St. Lawrence Island Inuit exhibit a “molar dominant” chipping pattern due to their emphasis on consuming tough and frozen food, while the two European samples show an “incisor‐dominant” pattern [42]. Dental chipping, unlike crown wear, is precipitated by accidents but may have a role in determination of population affinity by analyzing pattern of chipping and dietary habits of individuals from different regions.

## 7 Dental Maturity

Dental development, especially of third molar, is known to vary across populations and influences the approaches to both forensic age estimation and population affinity estimation [43]. It is found that Black British or other Black individuals develop third molars faster than White British individuals, which could have population‐specific implications relevant when determining the 18‐year legal threshold in forensic casework [43, 44]. It is shown that Moroccan, Turkish, Dutch Antillean, and Surinamese Creole children were found to achieve the dental milestones earlier than Dutch children, with African and Asian affinities linked to faster development and European affinities to slower development [44].

In forensic odontology, such variations complicate age estimation, risking misclassification (e.g., minors as adults) if universal models are used. Thevissen et al. [45] developed a Thai‐specific third molar database, improving age estimation accuracy for Thai individuals [45]. Dental maturity may supplement population affinity estimation alongside morphological traits, though variability requires caution. Standardized, population‐specific protocols can enhance forensic reliability.

## 8 Forensic Odontology Perspectives

This section explores forensic odontology perspectives on population affinity, integrating dental trait analyses with broader anthropological insights. Several studies have investigated the biological relationship between the Asian and Pacific Islander populations. For this purpose, the people from these regions have been physically and genetically examined. Unfortunately, the findings remain inconclusive. Some observations and examinations show that there are similar biological traits or features in East Asian and Southeast Asian populations [46, 47]; meanwhile, other examinations and observations highlight certain racial features that separate these two clusters [48]. It seems that it is inappropriate to generalize and consider all Asian people Mongoloids [48].

Although numerous studies have recognized East and Southeast Asian populations, the precise division differs depending on analysis method, and that there is no straight forward genetic connection between African and Australopapuan groups [23, 48, 49]. Dental evidence suggests Sundadonty or proto‐Sundadonty, which evolved in Southern Asia, may underline the origin of modern humans, and this pattern might have contributed to human colonization of Australia and the global population dispersal [50]. The Toba volcanic eruption in Sumatra (73,500 years ago) may have driven population movements from Southeast Asia, influencing dental trait distribution in Southern Asian, Pacific, and African populations [50].

Khamis et al. [15] analyzed dental crown variation in the four main population groups living in Malaysia. Dental casts obtained from 790 individuals were used to characterize variation in 13 dental crown traits within and between groups, and to assess affinities between them based on frequencies of occurrence of dental features [15]. The results showed similarities between Malays, Jahai (Negritos), and Chinese who conformed to Mongoloid Sinodont‐Sundadont dental patterns, while Indians conformed to an Indo‐European pattern [15]. Phenetic distance analysis showed that Indians were markedly separated from the other three groups, while Malays were closer to Jahai than to Chinese [15].

The dental complex historically referred to as “Mongoloid” dentition exhibits certain features, including shovel‐shaped incisors and torus mandibularis [51]. However, there remains considerable phenotypic variation in dental traits within and across Asian and Indigenous American populations associated with this broad grouping. In their 1951 comparative study, Moorrees and Darts analyzed tooth dimensions across Aleut, Japanese, Chinese, Javanese, Inuit, and Pecos Indian communities. Results revealed both commonalities in incisor sizes as well as inconsistencies among canines and premolars across groups [51]. The authors concluded that although dental patterns could be used to distinguish subgroups, caution should be exercised before ascribing a particular morphology or form with the racial category. Even after the lapse of several decades, the recommendation not to adopt an overly simplistic dental explanation that may have given rise to observations of the differences in the population is still relevant.

Dental traits like size and shape are highly inheritable and can exhibit marked variation across population. This could thus be a very useful tool in forensic identification. Edgar [52] conducted a study comparing the morphometry of teeth in a sample of 509 Americans grouped under different population affinities, in which statistically significant differences were revealed among the group [52]. Edgar then proceeded toward utilizing the findings of this study for building a logistic regression model that could classify population affinity based on dental characteristics alone. The models introduced have shown themselves very well when trying to approximate whether they belong to the European or African population group [52]. However, the equations produced less accurate results in identifying people of Hispanic descent. The study suggests that dental characteristics have potential in forensic racial identification. However, there are several limitations: the samples were drawn from a specific US population and therefore the results may not be replicated with other populations, dental features can vary depending on diet and environment; and the model was not 100% accurate which also means some room for error persists. These limitations show that caution should be taken when relying solely on dental traits for racial identification.

The study by Alsoleihat [53] presents a new method of predicting population affinity based on the frequency of certain dental traits [53]. The author's mean measure of divergence analysis was run on a Jordanian Arab sample and correctly classified population affinity in 84.31% of cases [53]. However, the technique also has its limitations– the method was able to classify population affinity into five broad groups: Western Eurasian, Sub‐Saharan African, Sino‐American, Sunda‐Pacific, or Australo‐Melanesian [53].

Caution, however, is needed to ensure accurate results. Dental analysis in conjunction with other forensic and anthropological techniques can provide additional evidence for population affinity estimation. The morphologic and morphometric analysis of teeth alone cannot estimate an individual's population affinity with complete accuracy; therefore, there is a need to refer to other sources of evidence [3, 4].

Observer training is essential for accurate population affinity estimation using dental traits. A study of 10 observers analyzing the same 9 plaques for 19 dental traits found low agreement on population affinity [54]. Agreement improved among experienced observers using simplified trait categories [54], but errors can compromise estimates. Estimating race from dental traits in forensic odontology risks reinforcing biological race misconceptions and perpetuating systemic racism in criminal justice [7, 55, 56]. Race, a social construct, does not reflect clinical biological variation, yet genomic data from genetic ancestry testing can reify race by linking genetic variations to racial categories [7, 56]. Racism, rooted in colonial ideologies, impacts health through structural inequities [7]. Many anthropologists advocate abolishing race‐based practices, applying critical race theory to reform methodologies and incorporate marginalized perspectives [55]. Population affinity estimates, based on continuous variation, offer a scientifically valid alternative, but require careful interpretation to avoid bias [7, 56].

## 9 Practical Applications in Forensic Context

Dental trait analysis is a promising in forensic contexts, but several pragmatic issues limit its medicolegal application. Although dental anthropology has developed many systems for traits and classification, their operational limitations often limit their usefulness in the forensic setting. Forensic cases are typically time‐sensitive, meaning the sample must be processed quickly, and as such, detailed morphological examination of the samples may not be workable. Many forensic laboratories operate without the requisite resources and specialized equipment for detailed dental analysis and often lack comprehensive comparative databases. A large disparity also exists in research methods developed in controlled academic settings and their practical application in actual field conditions. That is especially true in cases that need quick identification or that involve compromised remains. Classic division of people based on old racial typologies has always oversimplified genetic global diversity and never reflected the continuous and clinal nature of genetic and phenotypic variability [7]. The interpretation of dental traits outside a regional perspective is complicated by the increased mobility and admixture of populations in the modern world, and their inability to account for historical and modern genetic diversity [56]. Advances in anthropological genetics highlight the need for nuanced, population‐specific frameworks to enhance forensic applicability [56]. However, some strategies may improve the forensic applicability of dental trait analysis [7, 56]. These also encompass rapid standard assessment protocols, digital analysis tools, and comprehensive reference databases with contemporary population data. Recent advancement in forensic odontology focuses on quantitative analysis of dental structures [57]. Aschheim et al. derived a mathematical model using quadratic regression to define human maxillary dental arch class characteristics for a mean intercuspal distance of 33.8 mm and a parabola y = 0.040×^2^ − 0.0008x − 1.581 [57]. It had a greater precision with high predictive ability (R^2^ = 0.96), which offered an objective framework for forensic identification that surpassed traditional assessment methods [57]. Simon et al. [58] studied palatal geometry using intra‐oral scanner for sex discrimination and demonstrated an accuracy of 83.9%, which suggest a potential for population affinity estimation, warranting further exploration in forensic odontology.

Enhanced training programs and calibration exercises for forensic odontologists would significantly improve outcomes. Finally, clear documentation and reporting guidelines would allow such documents to meet the legal admissibility standard for prosecution without compromising methodology in medicolegal cases.

## 10 Conclusion

Dental anthropology enhances understanding of human population differentiation beyond race category. Although dental traits like shovel‐shaped incisors, Carabelli's cusp, and other morphological features can distinguish population differences, they cannot definitively determine ancestry. This review illustrates the complexities of human dental variation emphasizing that no single trait can establish population affinity.

In forensic use, it is clear that special attention must be paid to the data interpretation of dental traits. It requires multidisciplinary approaches, including genetic variability, environmental influences, and methodological limitations. Thus, the present work is designed to critically highlight the reliability of the historical application of dental traits in racial classification, and to contribute to similar, but finer and more scientifically justified investigations that demarcate the utility of the biological race concept.

It is recommended that future studies should focus on refining methods and expanding knowledge to improve the reliability and accuracy of results. The future of forensic odontology lies in comprehensive, technologically advanced approaches to estimate population affinity.

## Author Contributions

**Alok Atreya:** conceptualization, methodology, writing – original draft, writing – review and editing. **Ritesh G Menezes:** conceptualization, writing – review and editing, visualization, supervision. **Srinivasa Rao Bolla:** conceptualization, writing – review and editing. **Samarika Dahal:** conceptualization, writing – review and editing.

## Conflicts of Interest

The authors declare no conflicts of interest.

## Transparency Statement

The lead author Alok Atreya affirms that this manuscript is an honest, accurate, and transparent account of the study being reported; that no important aspects of the study have been omitted; and that any discrepancies from the study as planned (and, if relevant, registered) have been explained.

## Data Availability

Data sharing is not applicable to this article as no datasets were generated or analysed during the current study.
